# Hyperglycemia induces miR-26-5p down-regulation to overexpress PFKFB3 and accelerate epithelial–mesenchymal transition in gastric cancer

**DOI:** 10.1080/21655979.2022.2026730

**Published:** 2022-01-30

**Authors:** Xiaobo He, Xiao Cheng, Jianfeng Ding, Maoming Xiong, Bo Chen, Guodong Cao

**Affiliations:** a Department of General Surgery, First Affiliated Hospital of Anhui Medical University, Hefei, China; b Department of Pathology, Ningbo Diagnostic Pathology Center, Ningbo, China

**Keywords:** Hyperglycemia, miR-26, PFKFB3, epithelial–mesenchymal transition, gastric cancer

## Abstract

Gastric cancer (GC) is one of the most deadly malignancies with high morbidity worldwide. Cancer cells exhibited higher level of glucose catabolism than normal cells to meet the needs for rapid growth. Emerging evidences indicated that hyperglycemia has positive effects on the progression of tumor. As a vital regulator of glycolysis, 6-phosphofructo-2-kinase/fructose-2,6-bisphosphatase 3 (PFKFB3) was confirmed to have a higher expression level in tumor tissue and correlated with the prognosis of GC patients. However, the role of PFKFB3 in GC patients with hyperglycemia remains unclear. The data from The Cancer Genome Atlas (TCGA) and Gene Expression Omnibus (GEO) were utilized to analyze the expression level of PFKFB3 and conducted survival analysis of GC patients. Western blot assay was used to detect gene expression at the protein level. Small interfering RNA (siRNA) transfection assay was conducted to down-regulate the expression of PFKFB3. Cell functional assays were carried out to reflect the ability of cell proliferation and migration. The results indicated that PFKFB3 was significantly upregulated and its overexpression was associated with poor prognosis of GC patients. Besides, hyperglycemia stimulated the higher expression of PFKFB3 along with the enhanced proliferation, migration and epithelial–mesenchymal transition (EMT) in GC cells. Knocking down of PFKFB3 effectively reversed the effects of high glucose concentration on GC malignant phenotype and the opposite results were gained when miR-26-5p was inhibited. Therefore, PFKFB3 down-regulated by miR-26-5p inhibited the malignant phenotype of GC with hyperglycemia.

## Introduction

Gastric cancer (GC) is one of the most common human malignancies, ranking the third leading cause of cancer-related death globally [1]. It is widely known that reprogramming of glucose metabolism plays a vital role in cancer progression [2]. Cancer cells tended to performing glycolysis to provide adequate energy for fast cellular growth and proliferation even in the presence of oxygen, a phenomenon also known as ‘Warburg effect’ [3]. Diabetes mellitus (DM) is a common metabolic disorder syndrome with persistent hyperglycemia. Based on previous evidences, hyperglycemia promoted cell proliferation and metastasis in multiple cancers [4,5]. GC patients with hyperglycemia usually have poor prognosis [6]. Studies on the correlation between hyperglycemia and GC are still scare.

Glycolysis is the glycometabolic pattern of cancer cell. As a key regulatory factor in glycolysis, PFKFB3 could regulate the glycolysis process by changing the activity of PFK1, HK1 and other key enzymes [7]. The PFKFB3 protein is widely expressed especially in proliferative tissues, solid tumors and leukemia cells. Upregulation of PFKFB3 could be induced by mitogenic, inflammatory and hypoxia stimuli and the DNA synthesis phase of the cell cycle. In view of its significance in cancer metabolism, it is necessary to further study the function of PFKFB3 in diverse cancers [8]. PFKFB3 regulated glucose metabolism in tumor cells through a variety of signaling pathways. According to Telang S, the oncogenic Ras signaling pathway led to glycolysis shutdown and glioblastoma cell death by reducing PFKFB3 expression [9]. PFKFB3 was upregulated in multiple cancers, and PFKFB3 overexpression promoted the proliferation and metastasis of cancer cells [10,11]. Besides, PFKFB3 could also facilitate the chemoresistence and insensitivity of radiotherapy [12–14]. DM is one of the major public concerns worldwide, and some studies indicated that HIF1α/PFKFB3 pathways are abnormally activated in DM patients [15,16]. Therefore, we proposed a hypothesis that PFKFB3 expression can be upregulated with increasing glucose concentration and contributes to the poor prognosis of GC patients with hyperglycemia.

In recent years, more and more attentions were paid on the role of microRNA (miRNA) in cancer progression. As a kind of non-coding RNA, miRNA played a role in cellular differentiation and proliferation through combining with corresponding target mRNAs, resulting in mRNA translational inhibition or degradation [17]. The hyperglycemia-induced miR-26 expression via a HIF-dependent mechanism and the expression level of miR-26 was different in multiple cancers. The expression of miR-26 was significantly lower in breast cancer tissues than that in adjacent normal tissues, while its expression showed the opposite result in pituitary tumors [18,19]. Many publications has confirmed the role of multiple miRNAs in cancer progression, such as miR-26, miR-221 and miR-891 [20,21]. Previous studies indicated that miR-26 suppressed cell proliferation, enhanced cell chemosensitivity and promoted cell apoptosis in multiple cancers [22–25]. Besides, miR-26 was also confirmed to inhibit epithelial–mesenchymal transition [26]. However, few studies revealed its effects on GC patients with hyperglycemia.

In this study, miR-26 was screened out to be the most closely related miRNA of PFKFB3 by TargetScan (http://www.targetscan.org/vert_72/). In view of microRNA’s negative regulation of its target gene expression, we aimed to confirm that PFKFB3 down-regulated by miR-26-5p inhibited the proliferation and metastasis of GC induced by hyperglycemia. Firstly, we wanted to verify the prognostic value of PFKFB3 by analyzing the relationship between PFKFB3 and clinicopathologic parameters. Then we intended to detect the PFKFB3 expression under different glucose concentrations and perform siRNA transfection assay to test the function of PFKFB3. In general, we hypothesized that hyperglycemia induced the down-regulation of miR-26 to overexpress PFKFB3 and promote the malignant phenotype of GC cells.

## Methods

### Online databases

To compare the expression level of PFKFB3 in GC tissues with that in normal gastric tissues, gene expression profiling data was downloaded from TCGA (375 GC tissues and 32 normal tissues) and GSE118916 (15 GC tissues and 15 normal tissues). GSE84437 (433 GC tissues) was also downloaded from GEO (https://www.ncbi.nlm.nih.gov/geo/) to conduct gene correlation analysis between PFKFB3 and EMT-related markers. Moreover, survival analyses were performed to evaluate whether PFKFB3 expression was related to the prognosis of GC patients based on TCGA (https://portal.gdc.cancer.gov/) and Kaplan-Meier Plotter (http://kmplot.com) online databases. All data analyses were conducted with R programming language.

### Clinical samples

One hundred GC tissues and thirty adjacent normal tissues embedded with paraffin were collected from the First Affiliated Hospital of Anhui Medical University (Hefei, Anhui, China). These tissues were from GC patients who underwent radical resection between 2013.06 and 2014.06. They were followed up for at least 5 years and the survival status as well as clinicopathologic parameters were recorded. The informed consents from the patients were acquired, and this study was authorized by Biomedical Research Ethics Committee of Anhui Medical University.

### Immunohistochemistry

The histologic sections were primarily deparaffinized with xylene and ethanol with concentration gradients. Endogenous peroxidase blockage with 3% H_2_O_2_ and antigen retrieval using 0.01 mM citric acid buffer (PH: 6.0) in microwave oven were carried out. Sections were incubated in primary antibody PFKFB3 (1:100, Proteintech, USA) overnight at 4°C and incubated in the secondary antibody the next day for 30 minutes at room temperature. After the sections were washed with PBS, diaminobenzidine tetrahydrochloride (DAB) working solution was used to visualize PFKFB3 expression and hematoxylin was used to counterstain the sections.

The PFKFB3 expression level was assessed and scored independently by two senior pathologists according to the percentage of positive tumor cells (0, negative; 1, 0–25% positive; 2, 26%–50% positive; 3, 51%–75% positive; 4, 76%–100% positive) and the intensity of positive staining (0, negative; 1, weak; 2, moderate; 3, strong) in the field [27]. The final IHC score was acquired by multiplying the percentage of positive tumor cells and the intensity of positive staining. A scoring index ≥8 was considered high PFKFB3 expression, and a scoring index <8 was deemed as low PFKFB3 expression.

### Cell culture and transfection

Human gastric cancer cell lines MGC-803 and AGS purchased from the Chinese Academy of Sciences (Shanghai, China) were used in this study. Both of these two cell lines were cultured in RPMI 1640 medium (Procell, China) containing 10% fetal bovine serum, antibiotics (100 U/mL penicillin and 100 μg/mL streptomycin) and different volumes of sterilized glucose solution (200 g/L, Procell, China). The glucose concentrations of the complete medium were diluted to 5 mM, 15 mM and 25 mM, respectively, to construct euglycemia (5 mM) and hyperglycemia (15 mM, 25 mM) model in vitro. The medium was renewed daily to maintain the glucose concentration in each group, and cells after the eighth passage were used for subsequent experiments. All cells were maintained in a humidified atmosphere with 5% CO2 at 37°C.

siRNAs (including siPFKFB3-1, siPFKFB3-2 and siNC) and miRNA transfection primers (including miR-26-mimics-control, miR-26-mimic, miR-26-inhibitor-control and miR-26-inhibitor) were designed and synthesized by GenePharma (Shanghai, China). siRNA transfection assay and miRNA transfection assay were carried out with transfection reagent Lipofectamine 3000 (Invitrogen) referring to the recommended operational guidance. The detailed PFKFB3 siRNA and miRNA transfection primer sequences were as follows: siPFKFB3-1 forward: 5′-GGAGACACAUGAUCCUUCATT-3′, reverse: 5′-UGAAGGAUCAUGUGUCU CCTT-3′; siPFKFB3-2 forward: 5′-GCAUCGUGUACUACCUGAUTT-3′, reverse: 5′-AUCAGGUAGUACACGAUGCTT-3’; siNC forward: 5′-UUCUCCGAACGUG UCACGUTT-3′, reverse: 5′-ACGUGACACGUUCGGAGAATT-3′; miR-26-mimic-control:5′-CAGUACUUUUGUGUAGUACAA-3′; miR-26-mimic:5′-UUCAAGUAAUCCAGGAUAGGCU-3′; miR-26-inhibitor-control:5′-CAGUACUUUUGUGUAGUACAA-3′; miR-26-inhibitor:5′-AGCCUAUCCUGGAUUACUUGAA-3′.

### Western blot

As our laboratory described previously [6], the culture medium was discarded and cells were washed with phosphate buffer solution (PBS) three times. RIPA lysis buffer (Beyotime, China) was used to extract protein, and the BCA protein assay kit (Beyotime, China) was used to quantify protein concentration. The protein was separated with 10%–15% SDS-PAGE gel electrophoresis and transferred onto polyvinylidene fluoride membranes. Then, the membranes were immersed in PBST (phosphate buffered solution containing 0.1% Tween-20) with 5% skim milk powder for 1 h at room temperature to block nonspecific antigen. After being washed with PBST, the membranes were incubated with primary antibodies against PFKFB3 (1:1000, Proteintech, USA), vimentin (1:1000, Proteintech, USA), E-cadherin (1:1000, Proteintech, USA), N-cadherin (1:1000, Proteintech, USA), p-smad2/3 (1:1000, Proteintech, USA), smad2/3 (1:1000, Proteintech, USA), TGF-β (1:1000, Proteintech, USA), β-actin (1:1000, Proteintech, USA) for the whole night at 4°C. The membranes were washed with PBST three times the next day and incubated with the secondary antibodies (1:10,000, Proteintech, USA) for 1 h at room temperature. After being washed with PBST three times, the protein expression level in membranes can be detected with an enhanced chemiluminescence detection system (Amersham Imager 600, USA).

### Cell total number assay

The cell functional assays including cell total number assay, MTT assay, colony formation assay, wound-healing assay and migration assay were performed according to the protocols provided by the manufacturers. Cell counting was primarily carried out with cell counting plate. Cell functional assays, including cell total number assay, MTT assay, colony formation assay, wound-healing assay and migration assay, were conducted in this study. In cell total number assay, 1 × 10^5^ cells were planted into 6-well plates and the cell number in each group was counted for the next 5 days.

### MTT assay

In MTT assay, 2000 cells were planted into 96-well plates for 5 wells in each group and cultured for 72 h. After that, cells were incubated in 100 μl MTT reagent (mixture of 10 μl 5 mg/ml MTT and 90 μl cell culture medium) for 2 h. Then, the MTT reagent was discarded and 100 μl DMSO was added into each well in a dark environment. After 10 minutes, the relative optical density (OD) at 570 nm was measured with an automated plate reader (Bio-Rad, USA).

### Colony formation assay

In colony formation assay, 2000 cells were planted into 6-well plates and cultured for 14 days. Then, the medium was discarded and PBS was used to wash the cells. After fixed with 4% formaldehyde (200 μl/well) for 10 minutes, cells were immersed into 0.1% crystal violet (Sangon Biotech, Shanghai, China) for 10 minutes. Cells were washed with PBS and pictures were captured with an Olympus microscope (Olympus,Tokyo, Japan).

### Wound-healing assay

In wound-healing assay, cells were planted into 6-well plates and grew to 90% density. Then, 20 μl plastic tips were used to make linear wounds and cells were washed with PBS in each well. The width of wounds was measured, and pictures were captured with an Olympus microscope (Olympus, Tokyo, Japan).

### Migration assay

In migration assay, cells were planted into the upper chamber of 24-well plates with 200 μl serum-free medium at a density of 4 × 10^4^ cells/well. The lower chamber was added with medium containing 10% serum. After incubation for 24 h, the medium was discarded and the non-migrated cells were cleared off with a wet cotton swab. Then, the migrated cells were fixed with 4% formaldehyde for 10 minutes and dyed with 0.1% crystal violet (Sangon Biotech, Shanghai, China) for 10 minutes. The migrated cells were counted and pictures were captured with an Olympus microscope (Olympus, Tokyo, Japan).

### Rescue experiment

In order to further verify the regulatory effect of miR-26 on PFKFB3 along with its downstream pathways, rescue experiments were carried out in this study. siRNA transfection was performed in the control group; however, siRNA transfection and miRNA transfection were performed in the rescue group. Subsequently, the expression of PFKFB3 along with its downstream pathways and cell functional assays in two groups were compared.

## Statistical analysis

All results were repeated for at least three times, and the average was finally used in this study. The correlation of PFKFB3 expression and clinicopathologic parameters were analyzed using Pearson’s Chi-squared test in SPSS 19.0 (SPSS Inc., USA). Survival analyses were performed with survival package in R programming language. The results of cell functional experiments were analyzed with unpaired two-tailed t-tests. *P* < 0.05 was considered statistically significant.

## Results

In this study, the prognostic value of PFKFB3 was verified by analyzing the relationship between PFKFB3 and clinicopathologic parameters at first. Then we detected the PFKFB3 expression under different glucose concentrations and siRNA transfection assay was performed to test the function of PFKFB3. Finally, the possible upstream regulator, miR-26, was filtrated with TargetScan and its negatively regulatory effect on PFKFB3 was also confirmed.

### PFKFB3 upregulated in GC was correlated with poor prognosis and its expression was enhanced by hyperglycemia

**Figure 1.**
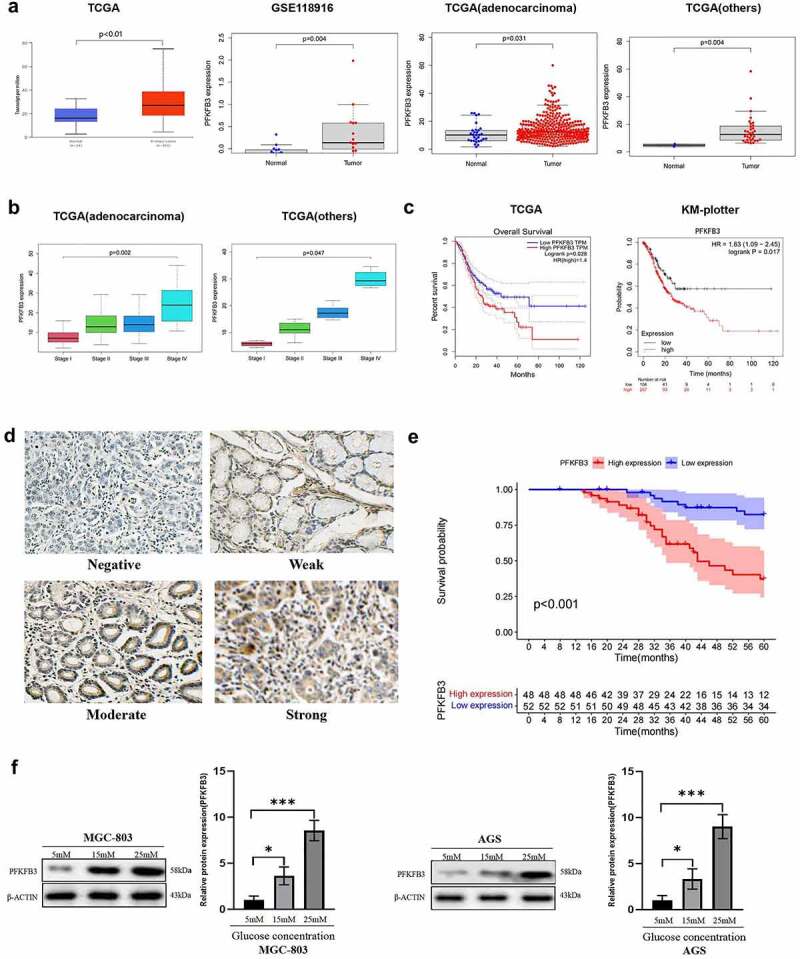
PFKFB3 overexpression was correlated with poor prognosis and its expression could be enhanced by hyperglycemia in GC. **(a, b, c)** PFKFB3 expression was significantly upregulated and its overexpression was correlated with clinical stage and the poor prognosis of GC patients based on online databases; **(d)** Representative IHC images with different PFKFB3 expression in GC tissues(×200 magnification); **(e)** PFKFB3 expression was significantly related to the overall survival of GC patients based on clinical samples;** (f)** Hyperglycemia could stimulate PFKFB3 overexpression in GC cells. *P < 0.05, **P < 0.01, ***P < 0.001

The mRNA expression data of PFKFB3 were extracted from TCGA and GEO (GSE118916) database to conduct bioinformatic analysis. As shown in Figure 1a, PFKFB3 expression was significantly upregulated in GC (TCGA: *P* < 0.01, GEO: *P* = 0.004). To further verify the results, we also conducted differential expression analysis of PFKFB3 on the subtypes of gastric tumors based on TCGA. The consistent results in subtypes of gastric tumors were gained (adenocarcinoma: *P* = 0.031, others (gastric cystic, mucinous and serous neoplasms): *P* = 0.004). Then we performed the correlation analysis of PFKFB3 expression and clinical stages in subtypes of gastric tumors based on TCGA. As shown in Figure 1b, the poor clinical stage was relevant to PFKFB3 overexpression (adenocarcinoma: *P* = 0.002, others: *P* = 0.047). Moreover, the results of survival analysis (Figure 1c) indicated that PFKFB3 overexpression was correlated with the poor prognosis of GC patients (TCGA: *P* = 0.028, KM plotter: *P* = 0.017). To further verify the results, immunohistochemistry staining was conducted to detect PFKFB3 expression in GC and adjacent normal tissues. 48/100 (48%) GC tissues presented high PFKFB3 expression, while 24/30 (80%) normal tissues showed low PFKFB3 expression (*P* = 0.006, Table 1). Representative images with different PFKFB3 expressions are exhibited in Figure 1d. The results of clinicopathologic correlation analysis indicated that PFKFB3 overexpression was significantly associated with higher blood glucose level (*P* = 0.018), larger tumor size (*P* = 0.006) and worse TNM stage (*P* = 0.027) (Table 2). Blood glucose (*P* = 0.039), TNM stage (*P* = 0.028) and PFKFB3 expression (*P* = 0.025) could act as independent prognostic factors for OS of GC patients according to the results of univariate and multivariate Cox regression analysis (Table 3). Overall survival (OS) analysis also indicated that GC patients with high PFKFB3 expression had a significantly shorter survival time than those with low PFKFB3 expression (*P* < 0.001, Figure 1e). Given the essential role of PFKFB3 in glucose metabolism, GC cell lines (MGC-803, AGS) were cultured in medium with different glucose concentrations (5 mM, 15 mM, 25 mM). The results of Western blot showed that the expression level of PFKFB3 increased with rising glucose concentration (figure 1f).

**Table 1.** The expression of PFKFB3 in GC and adjacent normal tissues

		PFKFB3 expression			
Group	n	High	Low	χ^2^	*P*
GC tissues	100	48	52	7.45	0.006*
Adjacent normal tissues	30	6	24		

* Statistically significant(*P*<0.05)

**Table 2.** Association of PFKFB3 expression with clinicopathologic parameters in GC patients

		PFKFB3 expression			
Parameters	n	High	Low	χ^2^	*P*
Age				0.188	0.664
<60	54	27	27		
≥60	46	21	25		
Gender				0.031	0.86
Female	53	25	28		
Male	47	23	24		
Blood glucose(mmol/L)				5.616	0.018*
<7	60	23	37		
≥7	40	25	15		
Tumor size(cm)				7.696	0.006*
<5	56	20	36		
≥5	44	28	16		
Lymph node metastasis				2.404	0.121
No	62	26	36		
Yes	38	22	16		
TNM stage				4.91	0.027*
I+ II	69	28	41		
III+IV	31	20	11		

* Statistically significant(*P*<0.05)

**Table 3.** Univariate and multivariate analysis of PFKFB3 expression with clinicopathologic parameters in GC patients

Parameters	Univariate analysisHR (95% CI)	*P*-value	Multivariate analysisHR (95% CI)	*P*-value
Age	0.728(0.359–1.476)	0.379		
Gender	0.631(0.311–1.279)	0.201		
Blood glucose(mmol/L)	3.142(1.539–6.414)	0.002*	2.312(1.041–5.133)	0.039*
Tumor size(cm)	1.975(0.980–3.981)	0.057		
Lymph node metastasis	2.319(1.151–4.670)	0.019*		
TNM stage	2.832(1.412–5.679)	0.003*	2.508(1.107–5.681)	0.028*
PFKFB3 expression	4.612(2.062–10.315)	<0.001*	2.736(1.135–6.595)	0.025*

* Statistically significant(*P*<0.05)

### PFKFB3 promoted proliferation and migration in GC cells

**Figure 2.**
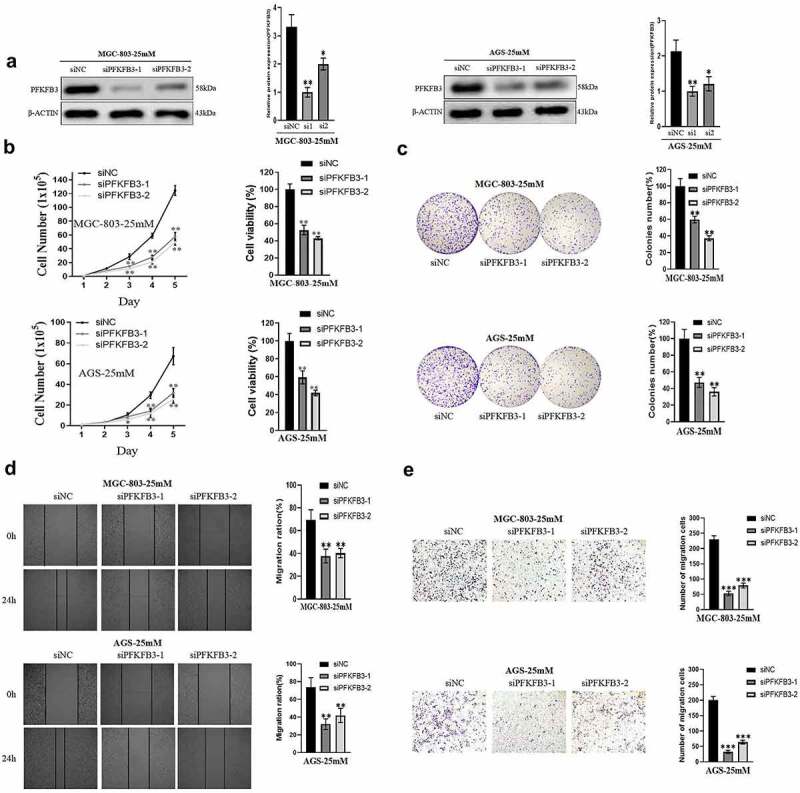
PFKFB3 knockdown suppressed the hyperglycemia-induced GC malignant phenotype in AGS and MGC803 cells. **(a)** Western blot analysis was conducted to confirm the transfection efficiency of PFKFB3; **(b, c)** The effect of PFKFB3 knockdown on proliferation was evaluated with cell total number assay, MTT assay and colony formation assay; **(d, e)** The effect of PFKFB3 silencing on migration was examined by wound healing and migration assays. *P < 0.05, **P < 0.01, ***P < 0.001

To investigate the role of PFKFB3 in GC, GC cell lines MGC-803 and AGS cultured in medium with 25 mM glucose concentration were transfected with siPFKFB3-1 or siPFKFB3-2. As shown in Figure 2a, the expression level of PFKFB3 decreased significantly after transfection in two cell lines. Then, cell functional assays, including cell total number assay, MTT assay, colony formation assay, wound-healing assay and migration assay, were performed. The results of cell total number assay and MTT assay indicated that the count and cell viability of two cell lines in siPFKFB3-1 or siPFKFB3-2 group were tremendously reduced in 5 days compared with siNC group (Figure 2b). Consistently, the cell colony number of two cell lines in siPFKFB3-1 or siPFKFB3-2 group was also dramatically decreased compared with siNC group (Figure 2c). To further study the effects of PFKFB3 knockdown on migration of GC cells, wound-healing assay and migration assay were subsequently conducted. As indicated in Figure 2d and Figure 2e, siPFKFB3-1 or siPFKFB3-2 effectively inhibited the migration of MGC-803 and AGS. Therefore, PFKFB3 promoted proliferation and migration of GC cells.

### PFKFB3 induced EMT and activated the TGF-β/Smad signaling pathway in GC cells

**Figure 3.**
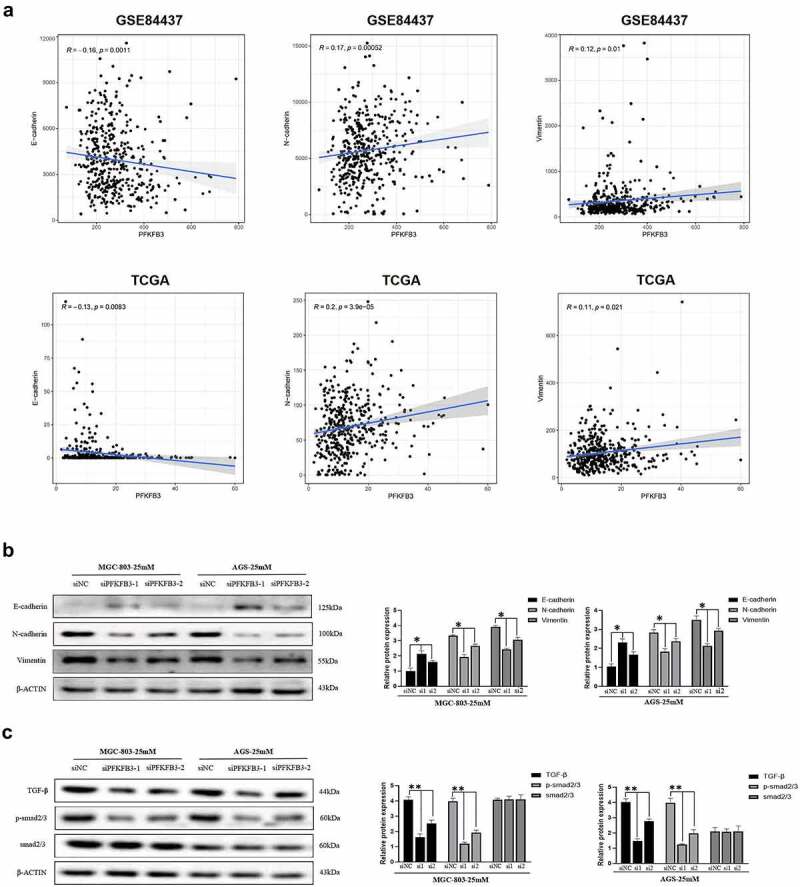
PFKFB3 silencing inhibited hyperglycemia-induced EMT and the TGF-β/Smad signaling pathway in AGS and MGC803 cells. **(a)** PFKFB3 expression was significantly correlated with EMT-related markers(E-cadherin, N-cadherin and Vimentin); **(b)** Western blot was performed to reflect the effects of PFKFB3 knockdown on EMT-related markers expression; **(c)** PFKFB3 knockdown was confirmed to inhibite the TGF-β/Smad signaling pathway with Western blot. *P < 0.05, **P < 0.01, ***P < 0.001

It is widely known that EMT plays an essential role in cancer metastasis and progression. To explore the possible relationship between PFKFB3 and EMT, gene correlation analysis was performed using data from GSE84437 and TCGA (gastric adenocarcinoma). As illustrated in Figure 3a, PFKFB3 was significantly correlated with three common EMT-related markers, E-cadherin (GSE84437: R = −0.16, *P* = 0.0011; TCGA: R = −0.13, *P* = 0.0083), N-cadherin (GSE84437: R = 0.17, *P* = 0.00052; TCGA: R = 0.2, *P < *0.001) and vimentin (GSE84437: R = 0.12, *P* = 0.01; TCGA: R = 0.11, *P* = 0.021). Consistently, the results of Western blot indicated that the expression of E-cadherin increased, while the expression of N-cadherin and vimentin decreased with PFKFB3 knockdown in both MGC-803 and AGS (Figure 3b). To further investigate the mechanism by which PFKFB3 promoted GC progression, GSEA was conducted to explore the possible downstream signaling pathway of PFKFB3. According to the results in Table 4, TGF-β/Smad was the most likely downstream signaling pathway of PFKFB3 with the maximum normalized enrichment score (TGF-β: 2.35, MAPK: 2.24, JAK-STAT: 2.19, WNT: 2.08). The results of Western blot equally revealed that the expression level of TGF-β and phosphorylated-Smad2/3 (p-Smad2/3) were reduced with PFKFB3 knockdown, while Smad2/3 was not significantly correlated with PFKFB3 (Figure 3c). Collectively, these results indicated that PFKFB3 promoted GC malignant phenotype via TGF-β/Smad signaling pathway.

**Table 4.** The downstream signaling pathway of PFKFB3 screened out by GSEA

GS	KEGG_TGF_BETA_SIGNALING_PATHWAY	KEGG_MAPK_SIGNALING_PATHWAY	KEGG_JAK_STAT_SIGNALING_PATHWAY	KEGG_WNT_SIGNALING_PATHWAY
**NES**	2.35	2.24	2.19	2.08
**NOM*p*-val**	<0.001	<0.001	<0.001	0.002

### Hyperglycemia induced PFKFB3 overexpression via miR-26-5p downregulation

**Figure 4.**
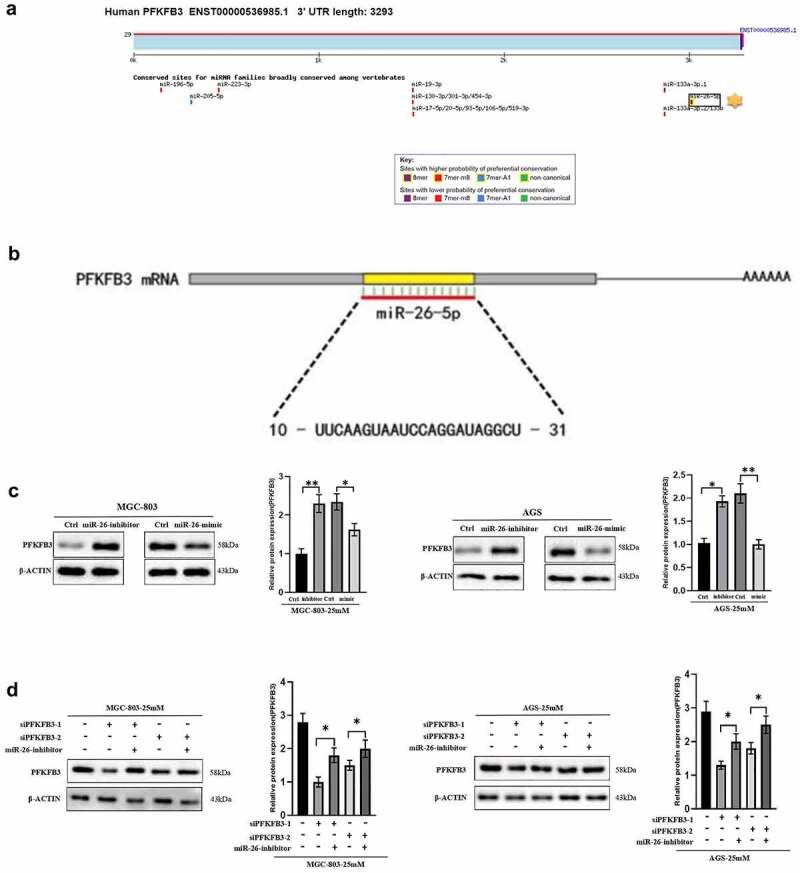
Hyperglycemia upregulated PFKFB3 expression by miR-26-5p downregulation. **(a)** Targetscan was used to explore the possible upstream regulators of PFKFB3(http://www.targetscan.org/vert_72/); **(b)** The sequence of miR-26-5p was illustrated; **(c)** miR-26-mimic and miR-26-inhibitor were used to explore the effects of miR-26 on PFKFB3 expression with Western blot; **(d)** Rescue experiment was conducted to further verify the role of miR-26 on PFKFB3 expression. *P < 0.05, **P < 0.01, ***P < 0.001

As a type of non-coding RNA, miRNA inhibited the expression of target genes through translational repression, mRNA degradation or mRNA cleavage. In this study, TargetScan database was used to screen out the possible upstream regulator of PFKFB3. As shown in Figure 4a, miR-26-5p was the most likely upstream regulator with the highest probability of preferential conservation on PFKFB3, and its sequence is illustrated in Figure 4b (10-UUCAAGUAAUCCAGGAUAGGCU-31). To confirm the effects of miR-26-5p on PFKFB3, miR-26-mimic and miR-26-inhibitor were used to transfect MGC-803 and AGS. The results indicated that the expression level of PFKFB3 was enhanced in GC cells transfected with miR-26-inhibitor, while miR-26-mimic reduced PFKFB3 expression (Figure 4c). Finally, the rescue experiment was performed to further verify the results. As shown in Figure 4d, miR-26-inhibitor partially counteracted PFKFB3 knockdown by siPFKFB3-1 or siPFKFB3-2 in both MGC-803 and AGS. In short, miR-26-5p inhibited PFKFB3 upregulation induced by hyperglycemia.

### miR-26-5p inhibited proliferation, migration, EMT and TGF-β/Smad signaling pathway in GC cells via PFKFB3 downregulation

**Figure 5.**
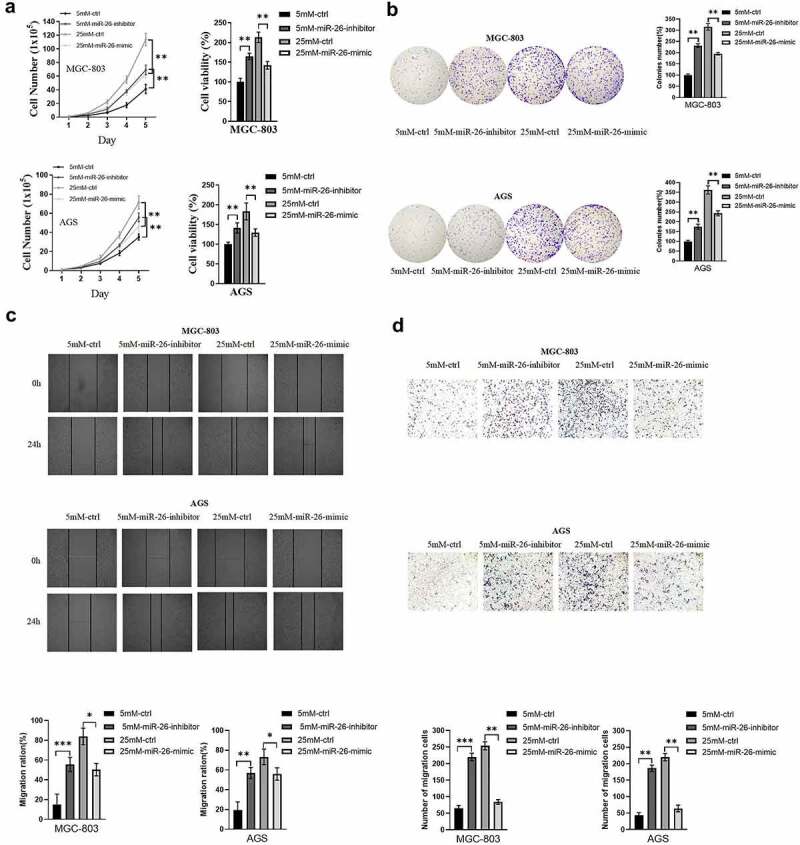
miR-26-5p inhibited hyperglycemia-induced GC malignant phenotype in AGS and MGC803 cells via PFKFB3 downregulation.** (a, b)** Cell total number assay, MTT assay and colony formation assay were conducted to evaluate the effects of miR-26-inhibitor or miR-26-mimic on proliferation in GC cells cultured in medium with different glucose concentrations(5mM and 25mM); **(c, d)** The effect of miR-26-inhibitor or miR-26-mimic on migration was examined by wound healing and migration assays in GC cells cultured in medium with different glucose concentrations(5mM and 25mM). *P < 0.05, **P < 0.01, ***P < 0.001

**Figure 6.**
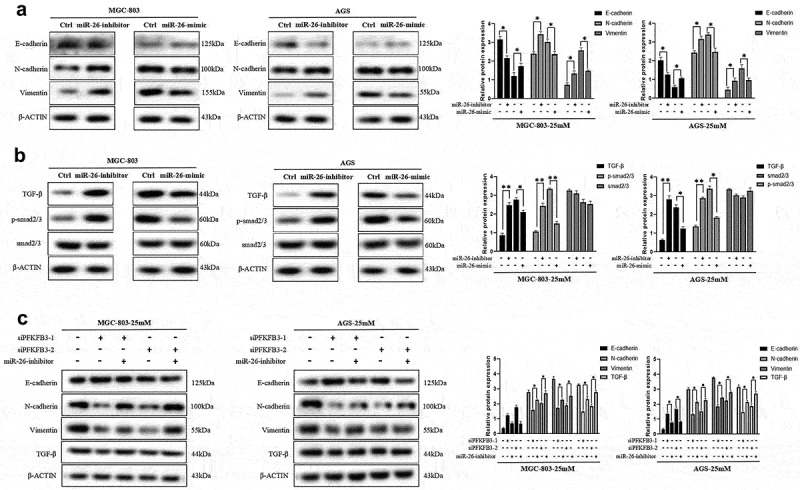
miR-26-5p inhibited hyperglycemia-induced EMT and the TGF-β/Smad signaling pathway in AGS and MGC803 cells. **(a)** The effects of miR-26-inhibitor or miR-26-mimic on EMT-related markers(E-cadherin, N-cadherin and Vimentin) expression was reflected with Western blot; **(b)** Western blot was performed to explore the effects of miR-26-inhibitor or miR-26-mimic on the TGF-β/Smad signaling pathway; **(c)** Rescue experiment was conducted to further verify the role of miR-26 on EMT and TGF-β/Smad signaling pathway. *P < 0.05, **P < 0.01, ***P < 0.001

To directly reflect the role of miR-26-5p on MGC-803 and AGS, cell functional assays of GC cells transfected with miR-26-inhibitor or miR-26-mimic were performed to compare with normal controls. GC cells cultured in medium with 5 mM glucose concentration were transfected with miR-26-inhibitor, while GC cells cultured in medium with 25 mM glucose concentration were transfected with miR-26-mimic. According to the results of cell total number assay, MTT assay and colony formation assay, the count, cell viability and colony number were significantly increased in GC cells transfected with miR-26-inhibitor and decreased in GC cells transfected with miR-26-mimic (Figure 5a, b). Consistently, miR-26-inhibitor promoted GC cell migration, while miR-26-mimic inhibited GC cell migration (Figure 5c, d). Moreover, miR-26-5p was correlated with the expression of EMT-related markers. As illustrated in Figure 6a, miR-26-inhibitor inhibited E-cadherin expression and promoted the expression of N-cadherin and vimentin, while the opposite effects were observed in GC cells transfected with miR-26-mimic. Similarly, miR-26-inhibitor activated TGF-β/Smad signaling pathway, and miR-26-mimic silenced TGF-β/Smad signaling pathway (Figure 6b). Finally, the rescue experiment was conducted to verify the results, the results indicated that miR-26-inhibitor counteracted E-cadherin upregulation and downregulation of N-cadherin, vimentin and TGF-β/Smad signaling pathway induced by PFKFB3 knockdown (Figure 6c). Therefore, miR-26-5p exerted a suppressive effect on the proliferation, migration, EMT and TGF-β/Smad signaling pathway in GC cells via PFKFB3 downregulation.

## Discussion

The poor prognosis of GC patients has been a huge public health concern due to metastasis, recurrence and drug resistance. More and more evidences indicated that tumor patients with hyperglycemia had worse prognoses than patients with euglycemia [28–31]. Hyperglycemia was reported to promote the acquisition of malignancy-associated properties including proliferation, migration, immune escape and EMT in pancreatic cancer [32–34]. Additionally, hyperglycemia reduced anticancer action of metformin and led to poor clinical outcomes in ovarian cancer [35]. Enhanced glycolysis played an essential role in cancer progression. As a key regulator in glycolysis, PFKFB3 promoted oncogenesis, proliferation and metastasis in multiple cancers by enhancing glycolysis process [36,37]. A recent study indicated that PFKFB3 was also a key effector protein in TGF-β/Smad signaling pathway, which involved in EMT of tumor cells [38]. Besides, PFKFB3 induced chemoresistance to protect cancer cells from apoptosis and blockage of PFKFB3 could effectively alleviate the malignant phenotype [39–41]. PFKFB3 inhibitor 3-(3-pyridinyl)-1-(4-pyridinyl)-2-propen-1-one (3PO) and its derivation 1-(4-pyridinyl)-3-(2-quinolinyl)-2-propen-1-one (PFK15) have been confirmed to inhibit glucose metabolism and exert potent antitumor activity in multiple cancers, especially gastric cancer [42,43]. Except for the effects on tumor cells, PFKFB3 also aggravated acute lung injury and pulmonary hypertension by upregulating endothelial glycolysis [44,45]. Our study mainly focused on the effects of PFKFB3 overexpression induced by hyperglycemia on GC progression and its mechanism. The results indicated that downregulation of miR-26-5p induced by hyperglycemia promoted GC cells proliferation, migration and EMT via PFKFB3 overexpression. Moreover, miR-26-5p upregulation or PFKFB3 knockdown could effectively reverse the malignant phenotype.

MicroRNAs (miRNAs) are a class of evolutionary conserved endogenous non-coding RNA who regulate gene expression by binding to the 3’-untranslated regions (3’-UTRs) of target mRNAs. According to previous evidences, miR-26 played an essential role in cell differentiation and closely correlated with the growth of normal tissues [46,47]. Notably, numerous studies have indicated that miR-26 inhibited proliferation, migration, invasion and induced apoptosis through different pathways in multiple cancers [25,48]. Besides, miR-26 also involved in substance metabolic process. According to a report, miR-26 suppressed adipocyte progenitor differentiation or fat production and provided novel potential therapeutic targets for obesity and related disorders [49]. Interestingly, the glucose concentration could regulate the expression of miR-26 and then affected GC phenotype in our study. As mentioned before, miR-26 was screened out as the upstream regulator of PFKFB3 and the results were further verified by Western blot. Therefore, the upregulation of PFKFB3 attributed to the miR-26 downregulation induced by hyperglycemia.

It is widely known that metastasis is closely related to cancer progression and the poor prognosis of patients. EMT, which is characterized by dissolution of epithelial cell–cell contacts and loss of apical-basal polarity, is an essential process in cancer metastasis [50]. Previous studies have confirmed that hyperglycemia enhanced metastasis by modulating the expression of EMT-related proteins through different mechanisms in several cancers [6,51]. This was consistent with the results of our study: hyperglycemia promoted EMT through PFKFB3 overexpression. As a traditional regulatory pathway, TGF-β has become a potent promoter in cancer progression through leading to immunosuppression and more importantly inducing EMT in multiple cancers [52]. Moreover, several reports also confirmed that miR-26 suppressed cancer malignant phenotype by downregulating TGF-β or inhibiting EMT [53,54]. In our study, TGF-β was identified as the downstream pathways of PFKFB3 through GSEA and the same result was acquired by Western blot. Consistent with other reports, miR-26 was also confirmed to significantly suppress TGF-β and EMT in GC. Summarily, these results proved that miR-26 inhibition induced by hyperglycemia could activate TGF-β signaling pathway and promote EMT in GC.

Our study confirmed the adverse effect of hyperglycemia on the prognosis of gastric cancer patients and first revealed that hyperglycemia promoted the malignant phenotype of gastric cancer cells via miR-26-5p/PFKFB3/TGF-β signaling pathway. This study might discover a novel therapeutic target for GC patients especially with DM. However, our study still has some limitations: First, it is not certain that GC cells cultured with hyperglucose medium could reflect the condition of GC cells in patients with hyperglycemia; Second, the expression level of miR-26-5p should be detected by PCR (Polymerase Chain Reaction) and dual-luciferase reporter assay should be conducted to further confirm the results; Third, hyperglycemic mice model should be constructed to further verify the results in vivo.

## Conclusion

In conclusion, our study indicated that PFKFB3 was correlated with clinicopathologic parameters and the prognosis of GC patients. Besides, hyperglycemia upregulated PFKFB3 expression via inhibiting the expression level of miR-26-5p, which promoted the proliferation, migration and EMT through the TGF-β/Smad signaling pathway in GC. Thus, preoperative management of hyperglycemia is of great significance to improve the prognosis of GC patients.

## Highlights

1. PFKFB3 overexpression induced by hyperglycemia is significantly correlated with the poor prognosis of GC patients.
2. Hyperglycemia promotes PFKFB3 expression via miR-26-5p down-regulation in GC cells.
3. PFKFB3 promotes the malignant phenotype and accelerates EMT in Gastric Cancer.

## Abbreviations

GC gastric cancer; PFKFB3 6-phosphofructo-2-kinase/fructose-2,6-bisphosphatase 3; miRNA microRNA; EMT epithelial–mesenchymal transition; GEO Gene Expression Omnibus; TCGA The Cancer Genome Atlas; TGF-β transforming growth factor β; DM diabetes mellitus
